# Supplementary material for: How massed practice improves visual expertise in reading panoramic radiographs in dental students: An eye tracking study
Source: PLoS One. 2020 Dec 3;15(12):e0243060. doi: 10.1371/journal.pone.0243060 (PMC7714201; doi:10.1371/journal.pone.0243060)
Supplement: Attachment — Submitted filename: Response to Reviewers.docx [file pone.0243060.s003.docx]

**PONE-D-20-05443R1
How massed practice improves visual expertise in reading panoramic radiographs in dental students: An eye tracking study
PLOS ONE
Response to Reviewers**

Dear Dr. Lanza, dear Reviewer,

we would like to thank the editor and the reviewer for the assessment of our manuscript and the feedback offered by the review. In the following, we will address the comments one by one. Reviewer comments are printed in regular font, responses in italics. Moreover, we have added sections from the manuscript whenever we made a change in it (in italics and quotation marks). We hope that this way we have addressed all issues raised by the reviewer in a satisfactory manner.

The authors

Comments from the Editor:

Dear Dr. Richter,

Thank you for submitting your manuscript to PLOS ONE. After careful consideration, we feel that it has merit but does not fully meet PLOS ONE’s publication criteria as it currently stands. Therefore, we invite you to submit a revised version of the manuscript that addresses the points raised during the second review process.

Please submit your revised manuscript by Dec 11 2020 11:59PM. If you will need more time than this to complete your revisions, please reply to this message or contact the journal office at plosone@plos.org. When you're ready to submit your revision, log on to https://www.editorialmanager.com/pone/ and select the 'Submissions Needing Revision' folder to locate your manuscript file.

Please include the following items when submitting your revised manuscript:

- A rebuttal letter that responds to each point raised by the academic editor and reviewer(s). You should upload this letter as a separate file labeled 'Response to Reviewers'.
- A marked-up copy of your manuscript that highlights changes made to the original version. You should upload this as a separate file labeled 'Revised Manuscript with Track Changes'.
- An unmarked version of your revised paper without tracked changes. You should upload this as a separate file labeled 'Manuscript'.

If you would like to make changes to your financial disclosure, please include your updated statement in your cover letter. Guidelines for resubmitting your figure files are available below the reviewer comments at the end of this letter.

If applicable, we recommend that you deposit your laboratory protocols in protocols.io to enhance the reproducibility of your results. Protocols.io assigns your protocol its own identifier (DOI) so that it can be cited independently in the future. For instructions see: [http://journals.plos.org/plosone/s/submission-guidelines#loc-laboratory-protocols](http://journals.plos.org/plosone/s/submission-guidelines#loc-laboratory-protocols)

We look forward to receiving your revised manuscript.

Kind regards,

Ezio Lanza, M.D.
Academic Editor
PLOS ONE

**5. Review Comments to the Author**

**Reviewer #1:**

Reviewer #1: Overall, the authors are to be commended for the efforts made to address the comments made by the reviewers.

*Thank you for this overall positive assessment of our revised manuscript and your constructive feedback.*

Regarding my comment on the inexistence of a control group, the authors argued that “it is unclear what the control group would be”. As an example, I might mention a study conducted by Andersen et al, which managed to compare the impact of massed and distributed practice in the learning curves of virtual mastoidectomy (Andersen SA et al. JAMA Otolaryngol Head Neck Surg. 2015; 141: 913-8).

Hence, as much as I understand that it may not be suitable to change the study design at this stage, it is, nonetheless, important that the authors acknowledge the weaknesses of the current approach.

*Thank you very much for this comment. We agree that it is necessary to acknowledge the weakness of our approach regarding the missing control group. We therefore addressed this aspect in the discussion section:*

*l.460*

*Also in radiology teaching and surgical skill training, studies have shown that spaced procedures are beneficial compared to massed practice [51,52]. Future research should therefore specifically compare massed and spaced practice for learning how to diagnose OPTs to identify potentials for further improvements of students’ accuracy. The present study did not aim at contrasting different training approaches because our focus was on the effects that current practice has on students’ skill development. Therefore, we implemented the present study in a within-subjects design within students’ regular training to achieve a high ecological validity. We acknowledge that this approach has its limitations due to a lack of a control group that would have been trained with a different approach.*

*(added) References:*

*52.	Andersen SAW, Konge L, Cayé-Thomasen P, Sørensen MS. Learning curves of virtual mastoidectomy in distributed and massed practice. JAMA Otolaryngol - Head Neck Surg. 2015;141: 913–918. doi:10.1001/jamaoto.2015.1563*

Furthermore, discussion-wise I suggest that the authors discuss the contextual interference hypothesis, which proposes that “when learning multiple skills, massing practice leads to better within-day acquisition, whereas random practice leads to better retention and transfer” (Savion-Lemieux T, Penhune VB.Exp Brain Res. 2010; 204: 271-81). In other words, the authors should discuss the potential variations of massed practice’s outcomes, depending on the task/learning objectives.

*Thank you for this suggestion. However, it is important to note that spaced vs. massed training is different from what has been proposed in research on contextual interference and interleaved practice, respectively. Spaced learning refers to the recommendation that when training a single skill, there should be pauses between multiple training sessions rather than training the same skill in a massed way. Thus, spaced vs. massed practice solely refers to the timing of the training. Contextual interference / interleaved practice refers to the training of multiple skills or skill components. Here it is recommended that these different skills are not to be trained one by one in a blocked fashion; rather, their training should be interleaved (abcabc etc.). It is true that in the case of interleaved practice this will mean that training of a single skill will be spaced at the same time. Nevertheless, the mechanism for the contextual interference effect are fundamentally different from that underlying spaced learning. In particular, interleaved practice is beneficial because it allows contrasting different training experiences, thereby highlighting that different contexts require application of different skills. The beneficial effects of interleaved practice only occur in delayed tests, whereas during training interleaved practice typically yields worse training performance. The confound between interleaved practice and spaced learning has been discussed and investigated, for instance, by Taylor and Rohrer (2009), who showed that interleaved practice has an effect above and beyond spacing. Because our study focused on spacing rather than interleaved practice, we have decided to not elaborate the concept of contextual interference in order to not cause any confusion of these two instructional principles. Rather, to highlight the focus of our paper on spaced learning, we added a footnote as follows:*

*l.470*

*Importantly, the current study refers only to the effects of massed practice regarding training of a single skill, where it has been suggested that training of that single skill should occur spaced in time. This recommendation is not to be confused with another instructional design principle, for which multiple labels have been used in the literature, that is, the contextual inference effect [53–55], the variability effect [56,57] or interleaved practice [58], respectively. Here it is suggested that when training multiple skills, these should be trained in an interleaved way (abcabcabc etc.) to highlight the differences between them. While interleaved practice of multiple skills results in spaced learning of the same skills, it is different from the focus of the present study.*

*(added) References:*

*553.	Battig WF. The flexibility of human memory. In: Lermak LS, Craik FIM, editors. Levels of processing in human memory. Hillsdale, NJ: Erlbaum; 1979. pp. 23–44.*

*54.	Shea JB, Morgan RL. Contextual interference effects on acquisition and transfer of a complex motor task. J Exp Psychol Hum PLearning Mem. 1979;5: 179–187.*

*55.	Brady F. A theoretical and empirical review of the contextual interference effect and the learning of motor skills. Quest. 1998;50: 266–293. doi:10.1080/00336297.1998.10484285*

*56.	Paas FGWC, Van Merriënboer JJG. Variability of worked examples and transfer of geometrical problem-solving skills: A cognitive-load approach. J Educ Psychol. 1994;86: 122–133. doi:10.1037//0022-0663.86.1.122*

*57.	Likourezos V, Kalyuga S, Sweller J. The Variability Effect: When Instructional Variability Is Advantageous. Educ Psychol Rev. 2019;31: 479–497. doi:10.1007/s10648-019-09462-8*

*58.	Taylor K, Rohrer D. The effects of interleaved practice. Appl Cogn Psychol. 2009;24: 837– 848. doi:https://doi.org/10.1002/acp.1598*
